# Splitting *Echinocactus*: morphological and molecular evidence support the recognition of *Homalocephala* as a distinct genus in the Cacteae

**DOI:** 10.3897/phytokeys.111.26856

**Published:** 2018-11-13

**Authors:** Mario Daniel Vargas-Luna, Patricia Hernández-Ledesma, Lucas Charles Majure, Raúl uente-Martínez, Héctor Manuel Hernández M cías, Rolando Tenoch Bárcenas una

**Affiliations:** 1 Laboratorio de Genética Molecular y Ecología Evolutiva, Facultad de Ciencias Naturales, Universidad Autónoma de Querétaro, Campus Aeropuerto, Querétaro, Querétaro 76140, México Universidad Autónoma de Querétaro Querétaro Mexico; 2 Instituto de Ecología A.C., Centro Regional del Bajío, 61600 Pátzcuaro, Michoacán, México Instituto de Ecología A.C. Pátzcuaro Mexico; 3 Department of Research, Conservation and Collections, Desert Botanical Garden, 1201 N. Galvin Parkway, Phoenix, Arizona 85008, USA Desert Botanical Garden Phoenix United States of America; 4 Departamento de Botánica, Instituto de Biología, Universidad Nacional Autónoma de México, 3er Circuito de Ciudad Universitaria, Del. Coyoacán, Ciudad de México, 04510, México Universidad Nacional Autónoma de México Ciudad de México Mexico

**Keywords:** Cactaceae, Cactaceae, HEA clade, morphological character evolution, North American Deserts

## Abstract

Molecular phylogenetic studies of the six currently accepted species in the genus *Echinocactus* have partially clarified certain aspects of its phylogeny. Most of the studies lack a complete sampling of *Echinocactus* and are based only in one source of data. Phylogenetic uncertainties in *Echinocactus*, such as the recognition of *Homalocephala* as a different genus from *Echinocactus*, the exclusion of *E.grusonii* or the affinities of *E.polycephalus*, are here resolved. Phylogenetic relationships of *Echinocactus* were reconstructed with a maximum parsimony, a maximum likelihood and a Bayesian approach including 42 morphological characters, four chloroplast markers (*atp*B-*rbc*L, *trn*H-*psb*A, *trn*L-*trn*F and *trnK/matK*) and two nuclear genes. The utility of these two nuclear regions related to the betalain cycles (DODA and *5GT*) are explored and discussed in relation to their potential as phylogenetic markers. Concatenated analyses with morphological and molecular data sets, plus 13 indels (2847 characters and 26 taxa), show general agreement with previous independent phylogenetic proposals but with strong support in order to propose the recognition of a reduced *Echinocactus* and the recognition of *Homalocephala* at the generic level. These results recovered a polyphyletic *Echinocactus* as currently defined. The here-named HEA clade, recovers the species of *Homalocephala*, *Echinocacuts* and *Astrophytum* as a monophyletic group with strong internal support. The *Homalocephala* (*H.texensis, H.parryi* and *H.polycephala*), was recovered as sister to the *Echinocactus* clade (*E.platyacanthus* and *E.horizonthalonius*), plus the *Astrophytum* clade. Consequently, we propose here to recognise a monophyletic *Echinocactus* and a monophyletic *Homalocephala* as two distinct genera with their own molecular and morphological synapomorphies. The evolution of some morphological characters supporting these clades are discussed, the necessary new taxonomic combinations for *Homalocephala* are proposed and an identification key for the genera, the species and the subspecies of the HEA clade are presented.

## Introduction

Current taxonomic delimitation of *Echinocactus* comprises six species of North American cacti (Barthlott and Hunt 1993; Anderson 2001, Hunt et al. 2006, Hunt 2016), *Echinocactusplatyacanthus*, *E.horizonthalonius*, *E.texensis*, *E.parryi*, *E.polycephalus* and *E.grusonii*. Four intraspecific taxa are also recognised, E.polycephalussubsp.xeranthemoides, E.polycephalussubsp.polycephalus, E.horizonthaloniussubsp.nicholii L.D. Benson and E.horizonthaloniussubsp.horizonthalonius (Coulter 1896, Benson 1969). Species of *Echinocactus* are distributed in all the North American desert regions, from the Tehuacán-Cuicatlán Valley in Puebla and Oaxaca, through the Chihuahuan, Sonoran and Mohave deserts, as well as into southern edges of the Great Basin Desert (Hernández and Gómez-Hinostrosa 2011). The species are characterised by having discoidal, globose to shortly columnar and strongly ribbed stems, large and elongated areoles that are divergent or confluent, a stem apex with a broad woolly crown, stout spines, short funnelform to campanulate apical flowers, flower and fruit areoles with pointed scales and covered with dense wool and large brown to black-brown seeds that vary from broadly oval to almost circular.

Britton and Rose (1922) based on differences in growth form and fruit morphology, segregated *E.texensis* Hopffer from *Echinocactus* proposing the monotypic genus *Homalocephala* Britton & Rose. Berger (1929) changed the rank of *Homalocephala* to a subgenus of *Echinocactus* in which he included only *E.texensis*. Bravo-Hollis and Sánchez-Mejorada (1991), based on the colour of the flower circumscribed the subgenusHomalocephala to include *E.texensis* and *E.horizonthalonius* Lem. Ferguson (1992) distinguished *Homalocephala* from *Echinocactus* by the stems which were rarely larger than 35 cm in diameter with sharp-edged ribs and discrete areoles, pericarpel and fruit with thick walls, pointed scales and trichomes scattered over the entire fruit wall, large keeled seeds with an indented hilum and testa cells surrounded by channelled anticlinal cell-boundaries. Ferguson believed that the unique morphological characters of *E.horizonthalonius* could support the creation of a new monotypic genus to include this species. Ferguson (1992) accepted in his study two subgenera, *Homalocephala*, including *E.texensis*, *E.parryi* Engelm., *E.polycephalus* Engelm. & J.M. Bigelow subsp. polycephalus and E.polycephalussubsp.xeranthemoides J.M. Coult. and subgenusEchinocactus including *E.platyacanthus* Link & Otto and *E.grusonii* Hildm.

The earlier restriction site variation study of Wallace (1995) was the only molecular phylogenetic analysis that included all six species of *Echinocactus*, accepted in contemporary taxonomic treatments. Wallace did not recover a monophyletic genus, since *E.grusonii* was recovered as sister to the *Astrophytum*-*Echinocactus* clade. The other five species were recovered in a single clade with two subclades, one including the type species, *E.platyacanthus* plus *E.horizonthalonius* and the second including *E.texensis, E.parryi, E.polycephalus* subsp. polycephalus and *E.polycephalussubsp.xeranthemoides*. Subsequent molecular studies (Butterworth et al. 2002, Bárcenas et al. 2011, Hernández-Hernández et al. 2011) have included only some species of *Echinocactus* and have also recovered a non-monophyletic genus, with *E.grusonii* more closely related to *Ferocactus.* The study of Bárcenas et al. (2011), based on the plastid *trnK/matK*, did not include *E.parryi* and recovered three of the five species within a clade and *E.grusonii* and *E.polycephalus* nested amongst other species of the Cacteae. The recovery of *E.grusonii* outside of core *Echinocactus* led to the proposal of a new monotypic genus named *Kroenleinia* Lodé to include *E.grusonii* as *Kroenleiniagrusonii* (Hildm.) Lodé (Lodé 2014). Nevertheless, the new genus *Kroenleinia* was not recognised in the most recent checklist of the Cactaceae and both *E.grusonii* and *E.polycephalus* were retained in the genus, plus no distinction between subgenus Echinocactus and *Homalocephala* was mentioned (Hunt 2016).

Most molecular systematic studies that have included species of *Echinocactus* have been limited to phylogenetic analyses based only on plastid datasets. Several studies have shown that DNA information, contained in some nuclear genes, can improve phylogenetic resolution at lower taxonomic levels due to higher nucleotide variability than that found in the cpDNA markers (Sang 2002). DODA and *5GT* are two nuclear genes that codify for key enzymes implicated in betalain synthesis in most families of Caryophyllales, including Cactaceae (Gandía-Herrero and García-Carmona 2013). Nucleotide sequences of these genes could be useful to generate a phylogenetic hypothesis comparable to those obtained with chloroplast markers and to perform combined analyses that could improve the phylogenetic signal. Likewise, the use of combined molecular and morphological datasets has shown its utility in detecting phylogenetic relationships not recovered in separated analyses (Nixon and Carpenter 1996). Recently, Sánchez et al. (2017), performing a combined analyses of morphological and molecular data resolved the phylogenetic relationships of some taxa in *Echinocereus* Engelm. (Cactaceae) that were not resolved in previous phylogenetic analyses conducted only with molecular evidence (Sánchez et al. 2014).

In this study, the phylogenetic relationships of all species and subspecies of *Echinocactus* are reconstructed based on DNA sequences of four chloroplast markers (*atp*B-*rbc*L, *trn*H-*psb*A, *trn*L-*trn*F and *trnK/matK*) and 42 morphological characters. In addition, the phylogenetic utility of two nuclear genes (DODA and *5GT*) in *Echinocactus* and outgroup taxa was explored. The topology obtained in a concatenated analysis, including molecular and morphological datasets, was used to trace the history of morphological characters. The goals of this study are to provide a phylogenetic hypothesis to clarify the position of *E.grusonii* and *E.polycephalus* by testing the monophyly of the currently accepted delimitation of *Echinocactus* and to test the taxonomic status of *Homalocephala*.

## Methods

### Taxon sampling

**Figure 1.**
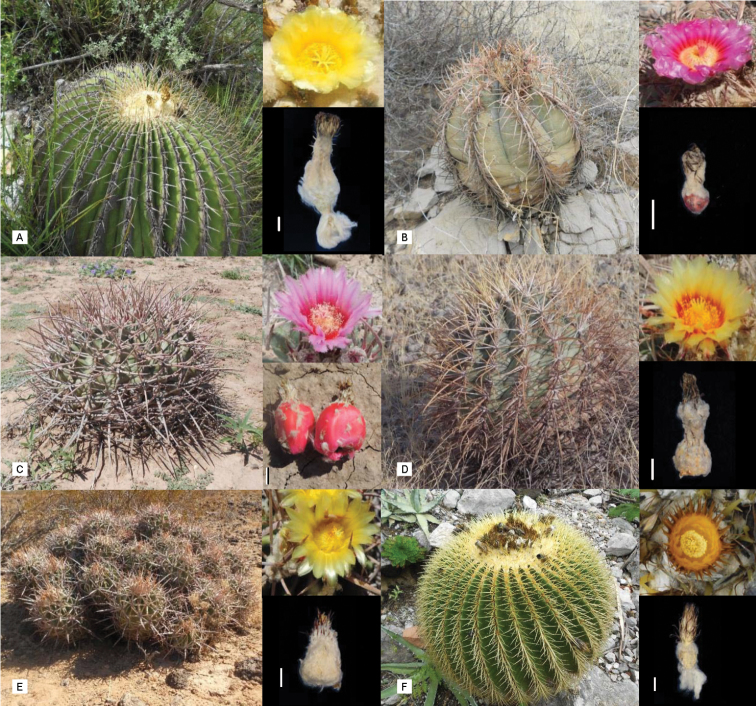
*Echinocactus* species. **A***E.platyacanthus* from Querétaro **B***E.horizonthalonius* from Chihuahua **C***H.texensis* from Chihuahua **D***H.parryi* from Chihuahua **E**H.polycephalasubsp.polycephala from Sonora **F***K.grusonii* from Querétaro. Line bar in fruit photographs is 1 mm.

A total of 26 taxa, including all six currently accepted species of *Echinocactus*, following Anderson (2001) and Hunt et al. (2006), (Fig. 1) are included in this study. DNA sequences from other genera such as *Astrophytum, Aztekium* Boed., *Geohintonia* Glass & W.A. Fitz-Maur. and *Ferocactus* from Cacteae and *Carnegieagigantea* (Engelm.) Britton & Rose, from Phyllocacteae were generated and used as outgroups. When possible, three terminals for each *Echinocactus* species, representing individuals from different populations or in some cases subspecies, were included (Appendix 1). Samples were collected from wild populations under Mexican and Arizonian permits, from herbarium specimens at ASU, DES, QMEX and MEXU and from the living collection of the Desert Botanical Garden, which also originated from wild collected specimens (Appendix 1). Additionally, 41 nucleotide sequences deposited in GenBank (www.ncbi.nlm.nih.gov/Genbank) were downloaded and included in the analyses. *Opuntiaficus-indica* (L.) Mill. from subfamily Opuntioideae was used as a functional outgroup for concatenated analyses and for most of the non-concatenated analyses, except for those conducted with the nuclear genes, in which, in order to have a wider sampling, we included *Amaranthustricolor* L. and *Beta vulgaris* L. of Amaranthaceae. For nuclear DNA analyses, *A.tricolor* was used as the functional outgroup.

### DNA isolation, PCR and sequencing

Total genomic DNA was extracted from silica gel dried stem tissues or from herbarium samples. Approximately 20 to 30 mg of tissue was ground, from which DNA was extracted with a CTAB 2% protocol described in Doyle and Doyle (1987) and as modified by Bárcenas (2015). We also used previously extracted DNA preserved in TE 10 mM, pH 8.0, deposited in the collection of the Laboratorio de Genética Molecular y Ecología Evolutiva of the Universidad Autónoma de Querétaro, México. The *atp*B-*rbc*L intergenic spacer was amplified with the primers of Demesure et al. (1995). The amplification parameters were: 96 °C for 5 min; 35 cycles of 94 °C for 50 s, 47 °C for 1 min, 72 °C for 1.5 min and a final extension of 72 °C for 10 min. Primers “E” and “F” designed by Taberlet et al. (1991) were used for the amplification of the *trn*L-*trn*F intergenic spacer. PCR parameters to amplify the region were: 96 °C for 5 min; 35 cycles of 94 °C for 1 min, 56 °C for 1 min, 72 °C for 1.5 min and a final extension of 72 °C for 5 min. The primers designed by Hamilton (1999) were used to amplify the *psb*A-*trn*H intergenic spacer. PCR parameters for the *psb*A-*trn*H were: 96 °C for 4 min; 35 cycles of 94 °C for 1 min, 48 °C for 1 min, 72 °C for 1.5 min and a final extension of 72 °C for 5 min. Primers used to amplify the two nuclear genes were designed by Felker et al. (2008). The best PCR conditions for DODA were 96 °C for 5 min; 35 cycles of 94 °C for 45 s, 54 °C for 1 min, 72 °C for 1.5 min and a final extension of 72 °C for 5 min. The most successful conditions for *5GT* were 96 °C for 5 min; 35 cycles of 94 °C for 45 s, 52 °C for 1.5 min, 72 °C for 1.5 min and a final extension of 72 °C for 4 min. PCR products were commercially purified and sequenced in both directions using the same primers in Macrogen Inc., Seoul, South Korea or at the ASU DNA Laboratory, Tempe, Arizona. The 22 sequences of *trnK/matK*, included in the analyses, were downloaded from GenBank (Appendix 1).

### Morphological characters

A set of 42 morphological characters were coded (Appendix 2) by observations during field work and by examination of herbarium specimens generated in this study and specimens already deposited in herbaria (ASU, DES, MEXU and QMEX). Primary homology statements (Hawkins et al. 1997) were postulated following the criteria of conjunction and similarity of Patterson (1982) and De Pinna (1991). To code different states of plane figures and three-dimensional shapes, we adopted the terminology published by Radford (1974) and Eggli (1993) and constructed a binary and multistate morphological character matrix in Mesquite 3.02 (Maddison and Maddison 2015). Six characters were coded from seedling stage or during the first year of development. The seedlings were cultivated from seeds collected during fieldwork or commercially bought from nurseries. All seedlings were grown under the same environmental conditions on campus without tracking temperature, light or humidity. Certain morphological characters such as shape of ribs and shape of flowers were also obtained from bibliographic sources (Bravo-Hollis and Sánchez-Mejorada 1991, Ferguson 1992, Chamberland 1997, Barthlott and Hunt 2000, Doweld 2000, Hunt et al. 2006) only for some of the outgroup taxa in *Ferocactus*. The seed size categories proposed in Barthlott and Hunt (2000) were used to assign character states. To ensure that we assigned the correct category, we measured ten random seeds per species and the average was used as the seed size indicator for each of the categories. Character state delimitations for the number of spines per areole were based on the counts of 25 areoles per species from five different plants and five different areoles of at least two different populations for the majority of the species except for *Opuntiaficus-indica* (n=1 areole, one plant, one population), *Carnegieagigantea* (n=5 areoles, one plant, one population), *Echinocactushorizonthalonius* (n=13 areoles, three plants, two populations) and *Astrophytumasterias* (n=15 areoles, three plants, one population). The age of the plants varied from 1 to 12 months and no change in the number of spines per areole was detected in that period of time, that is, plants of one month had the same number of spines that plants of 3, 4 or 12 months old. No plants older than 12 months were included in this delimitation. The average of spine number per seedling was calculated in order to reduce the effect of pseudoreplication (counting different areoles of the same seedling) and, from 421 counts, only 88 observations were included in the statistical analysis as unique events. The hypothesis that three groups existed and were statistically different was confirmed with a Kruskal and Wallis test and a post-hoc test for pairwise multiple comparison (Nemeyi’s test) in R with the library PMCMR (Pohlert, 2014). The groups were then defined as state (0) from 1 to 4 spines per areole; state (1) from 5 to 8 spines per areole and state (2) from 9 to 12 spines per areole as defined in Appendix 2.

### Alignments and phylogenetic analyses

In order to generate consensus sequences, both forward and reverse sequences were assembled manually in PHYDE (Müller et al. 2007) and checked against each of the chromatograms to resolve potential ambiguities. Newly generated sequences were blasted in GenBank to ensure that they corresponded to their respective regions, thus avoiding the use of contaminated sequences. Based on the micro-architectural characterisation of the *psb*A-*trn*H intergenic region (IGR) for the Cactaceae proposed by Hernández-Ledesma and Bárcenas (2016), the intergenic spacer (IGS) was excluded since it is a highly variable mutational hot-spot making alignment highly subjective. The DODA forward and reverse sequences were impossible to assemble and thus precluded the generation of consensus sequences. This probably was due to the presence of an intron located at position 199 to 960 as annotated in the sequences of *Opuntia-ficus-indica* (GenBank accessions EU089741 and EU089742). Thus the aligned matrix of DODA was constructed by simple concatenation of the forward and reverse sequences for each of the terminals.

Consensus sequences were automatically aligned with MUSCLE (Edgar 2004), as implemented in MEGA 7 (Kumar et al. 2015) and then manually corrected in PHYDE, reducing the number of gaps and increasing the match amongst nucleotides. Three concatenated matrices were constructed in WINCLADA 1.00.08 (Nixon 2002), one included only the four chloroplast markers (here named the cpDNA matrix); the second included the cpDNA matrix plus the 42 morphological characters and 13 indels (cpDNA plus morphology matrix); the third matrix included the cpDNA plus the morphological matrix and the sequences of the two nuclear genes (total evidence matrix). The lack of several sequences for DODA precluded the analysis of the complete dataset for both of the nuclear genes and only the tree for 5GT is shown in Figure 5. The 13 indels were coded from the *atp*B-*rbc*L marker and were considered as additional presence/absence characters.

For each individual dataset and for the cpDNA concatenated matrix, maximum parsimony (MP) and maximum likelihood (ML) analyses were performed in MEGA 7, while Bayesian analyses (BA) were carried out in MRBAYES 3.2.6 (Huelsenbeck and Ronquist 2001). The cpDNA plus morphology and the total evidence matrices were analysed only by MP and Bayesian methods, since no partition for molecular and morphological characters could be set for the ML analysis. The MP analyses for the cpDNA plus morphology and total evidence matrices were done in PAUP 4.0 (Swofford 2002). The best-fit evolutionary models for all matrices were selected according to the Bayesian Information Criterion (BIC) implemented in MEGA 7. ML analysis for the cpDNA matrix was run with the GTR+G evolutionary model. BA analysis of the cpDNA matrix was run setting the following partition and evolutionary models: *atp*B-*rbc*L (GTR+G), *psb*A-*trn*H and *trnK/matK* (HKY+G) and *trn*L-*trn*F (HKY). The BA analyses of cpDNA plus morphology and the total evidence matrices were performed with the same settings as the cpDNA matrix, but for the total evidence matrix the models selected for the DODA and *5GT* nuclear genes were K2 and K2+G, respectively. Morphological characters and indels in BA analyses were run under the Mkv model and coded as variable. For cpDNA concatenated analyses, we tried when possible to sample the same three individuals for the ingroup; nevertheless, some terminals were assembled by combining sequences from different individuals, as extracted DNA was exhausted and no more tissue existed for other extractions.

MP analyses were run with heuristic searches, TBR, random addition of sequences and 1000 replicates. A 50% majority rule consensus tree was calculated from the most parsimonious trees. ML analyses were run with an initial neighbour-joining tree with the bionNJ algorithms for the heuristic search and then by selecting the tree with the best log likelihood value. Support of nodes for the MP and ML consensus tree was estimated from 1000 non-parametric bootstrap replicates and by setting the same heuristic search parameters as implemented in MEGA 7 and PAUP 4.0. The BA analyses were performed with two runs of four chains and 10,000,000 iterations, sampling every 5000 generations. The value of the average standard deviation of split frequencies (Lakner et al. 2008) was used as an indicator of convergence when it reached 0.002 for the cpDNA, 0.001 for the cpDNA plus morphology and 0.002 for the total evidence analyses. A 25% burnin fraction of trees was set and the remaining trees were summarised in a majority rule consensus tree, in which the posterior probabilities were calculated and used as indicators of support for the clades.

### Character optimisation and character history

To identify putative synapomorphies and homoplasies for each of the recovered clades, an unambiguous character optimisation on an MP strict consensus tree created with the total evidence matrix was conducted in WINCLADA 1.00.08. Acctran and deltran optimisations were explored in WINCLADA to identify additional synapomorphic characters. Since the optimisation analyses identify several characters as homoplastic, they create a very large tree which could not be edited for publishing purposes, thus we only show the synapomorphic characters. A character history was inferred using the resulting topology of the BA analysis of the total evidence matrix, in which ancestral character states for nodes were estimated using the parsimony approach implemented in MESQUITE 3.02.

## Results

### Sequences and character matrices

In this study, 34 new sequences from the a tpB-*rbc*L were generated and deposited in GenBank (accessions MH129808−MH129841), 32 of the *psb*A-*trn*H (accessions MH129842−MH129873), 10 from *trn*L-*trn*F (accessions MH138282−MH138291), 33 from *5GT* (accessions MG149503−MG149535) and 17 from DODA (accessions MG149536−MG149552). Due to the presence of several indels, the newly generated *atp*B-*rbc*L sequences varied considerably in length, from 385 to 782 nucleotides. New sequences of the other molecular markers were less variable in size and no considerable indels were detected. The sequences of the *psb*A-*trn*H (including the IGS) ranged from 241 to 236 nucleotides. The length variation of the *trn*L-*trn*F sequences was from 801 to 826. The newly generated partial DODA sequences ranged from 290 to 330 nucleotides long and the 33 sequences of the *5GT* ranged from 469 to 597. Aligned sequences for the three concatenated datasets were 1855 nucleotides long for the cpDNA matrix, 1910 characters for the cpDNA plus morphology matrix and 2847 for the total evidence matrix. Table 1 shows a statistical summary for each of the individual and for the concatenated matrices. Alignments and trees are available in TreeBASE (https://www.treebase.org/) submission 23037.

**Table 1.** Summary statistics of the datasets analyzed.

	Total length (nucleotides)	Informative characters	Terminals	Taxa
***atp*B-*rbc*L**	913	76 (8.3%)	48	28
***psb*A-*trn*H**	319	48 (15.04%)	47	24
***trn*L-*trn*F**	1082	62 (5.7%)	48	21
***mat*K**	1292	43 (3.3%)	30	24
** DODA **	333	73 (22%)	23	16
***5GT***	610	231 (38%)	37	21
**cpDNA**	1855	103 (5.5%)	35	26
**cpDNA + morphology**	1910	134 (7.0%)	26	26
**Total evidence**	2847	207 (7.3%)	26	26

### Phylogenetic analyses

**Figure 2.**
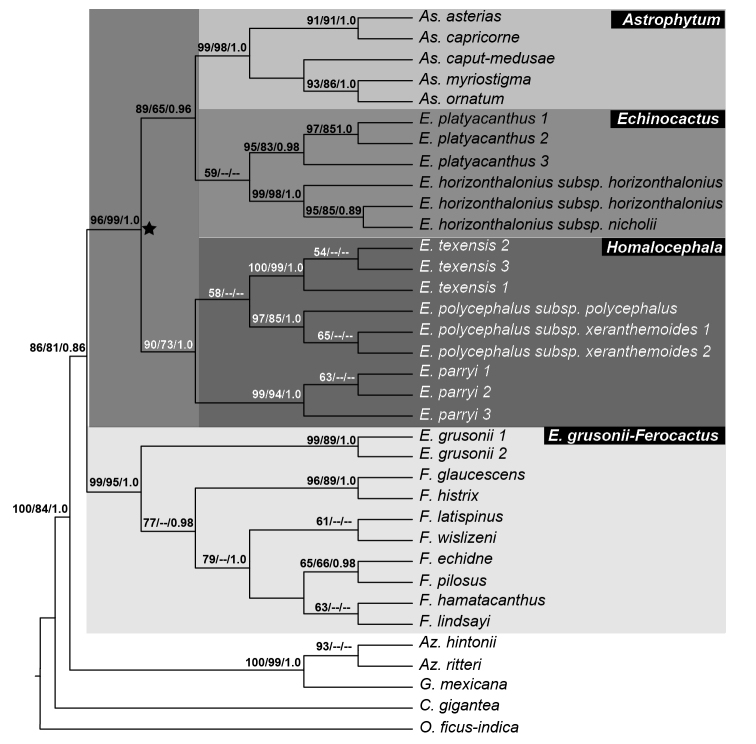
Phylogenetic relationship of *Echinocactus* inferred with the cpDNA matrix. Maximum parsimony 50% majority rule consensus tree of 4 most parsimonious trees of 347 steps with a consistency index of 0.681 and retention index of 0.848. Values in nodes correspond (from left to right) to maximum parsimony bootstrap (mpb), maximum likelihood bootstrap (mlb) and posterior probabilities (pp). The black star indicates the HEA clade with three subclades: *Astrophytum, Echinocactus* and *Homalocephala* highlighted.

**Figure 3.**
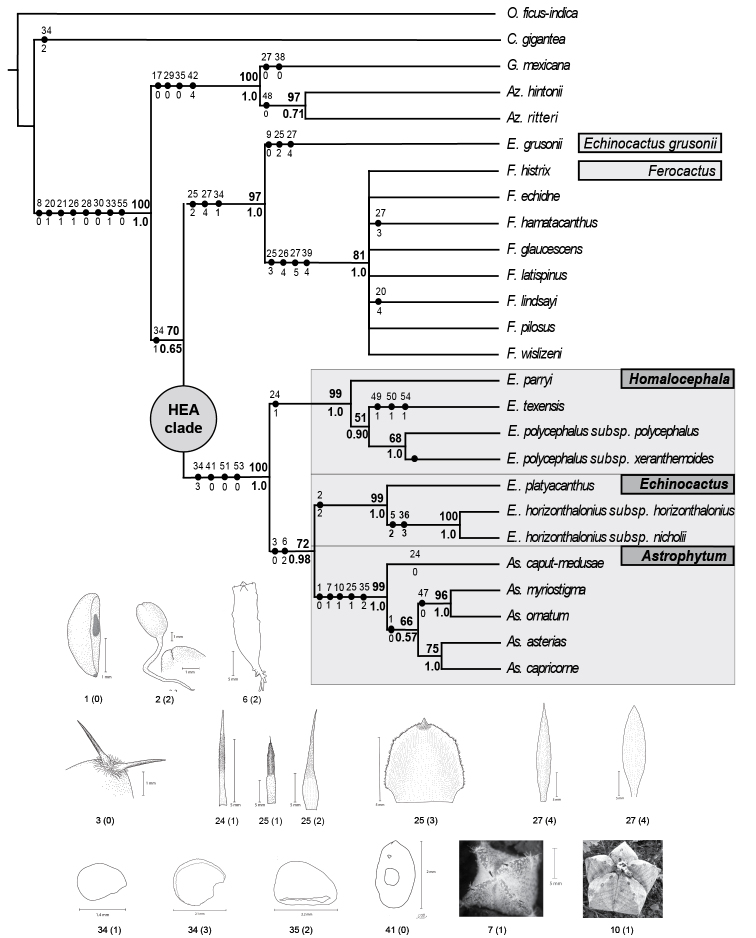
Phylogenetic relationships of *Echinocactus* inferred with the total evidence matrix. Maximum parsimony strict consensus tree of 2 most parsimonious trees of 863 steps with a consistency index of 0.790 and a retention index of 0.770. Values above/below nodes indicate maximum parsimony bootstrap (mpb) and posterior probabilities (pp), respectively. Black circles indicate synapomorphic characters identified by the character optimisation analyses. Numbers above/below circles indicate characters and character states, respectively (see Appendix 2). The HEA clade is labelled showing the three genera: *Astrophytum*, *Echinocactus* and *Homalocephala*. Line drawings illustrate the morphological synapomorphies for respective clades.

**Figure 4.**
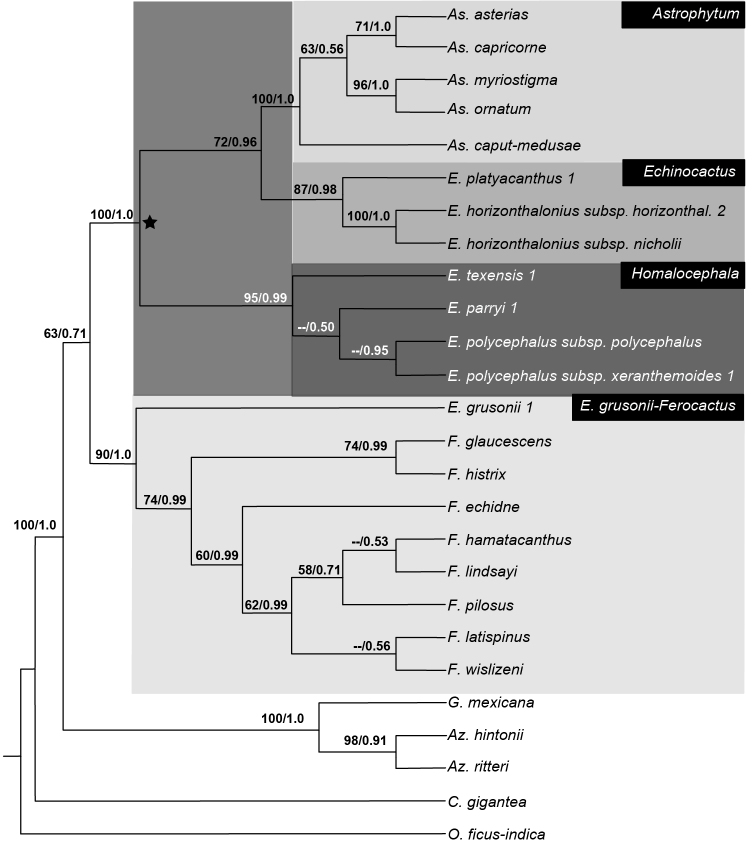
Phylogenetic relationship of *Echinocactus* inferred with cpDNA plus morphology. Bayesian 50% majority rule consensus tree. Values in nodes correspond (left to right) to bootstrap values from maximum parsimony (mpb) and posterior probabilities (pp). The black star indicates the HEA clade and its three subclades *Astrophytum, Echinocactus* and *Homalocephala* highlighted.

**Figure 5.**
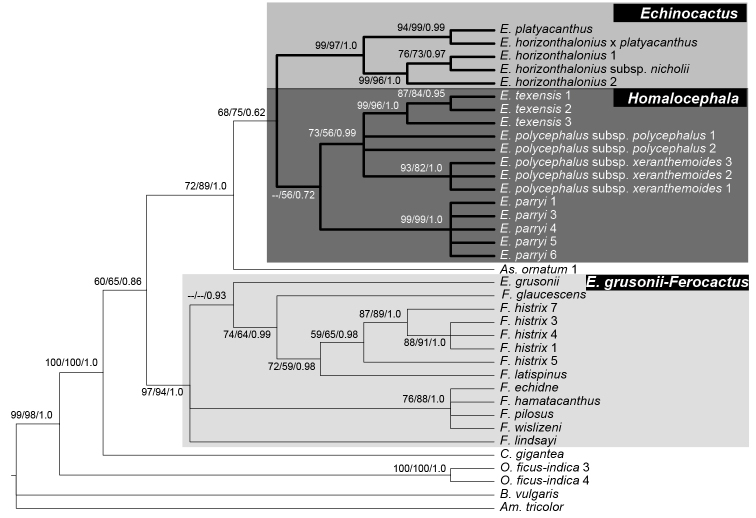
Bayesian 50% majority rule consensus tree showing phylogenetic relationship of *Echinocactus* inferred with 5*GT*. Values in tree nodes correspond (left to right) to bootstrap values from maximum parsimony (mpb), maximum likelihood (mlb) and posterior probabilities (pp). The clades highlighted in dark and light grey correspond to the *Echinocactus*, *Homalocephala* and *K.grusonii-Ferocactus* clades.

**Polyphyletic *Echinocactus***. Results obtained with the three concatenated matrices recovered a polyphyletic *Echinocactus. E.grusonii* was recovered as sister to *Ferocactus* (hereafter the *K.grusonii-Ferocactus* clade) with strong support in all three concatenated analyses and also in the *5GT* nuclear analyses (Fig. 5). Support values for this clade in the cpDNA analysis were 99% for maximum parsimony bootstrap (mpb), 95% for maximum likelihood bootstrap (mlb) and 1.0 of posterior probability (pp) as shown in Figure 2. The cpDNA plus morphology and the total evidence analyses also recovered the *K.grusonii*-*Ferocactus* clade with strong support, (90% mpb and 1.0 pp) and (97% mpb and 1.0 pp), respectively (Figs 3, 4).

The cpDNA plus morphology and the total evidence analyses recovered *Echinocactus* in two strongly supported clades. One clade included *E.texensis, E.parryi* and *E.polycephalus* (here after the *Homalocephala* clade) while the other clade included *E.platyacanthus* and *E.horizonthalonius* (here after the *Echinocactus* clade). These two clades, together with the *Astrophytum* clade, were recovered with high support as a monophyletic group (here after the HEA clade), (Figs 2, 3). The HEA clade was previously recovered in several phylogenetic studies (Wallace 1995, Butterworth et al. 2002, Vázquez-Lobo et al. 2015) and also has been recognised as a taxonomic group by some authors (Berger 1929, Benson 1982).

**HEA Clade.** This clade was recovered with the highest support by the cpDNA and morphology and the total evidence analyses (Figure 3, 4), respectively. This clade is also supported by two deletions found in the *atp*B-*rbc*L dataset, the first deletion being located at position 390 to 394 and the second at position 449 to 457 of the alignment.

All concatenated analyses recovered three major clades within the HEA clade. One clade included the clade of *E.texensis, E.parryi* and *E.polycephalus*, (*Homalocephala* clade), the second clade included *E.platyacanthus* and *E.horizonthalonius* (*Echinocactus* clade), which was recovered with high support and as sister to the third clade, the *Astrophytum* clade (Figs 2, 3). However, the ML and BA analyses of the cpDNA matrix did not recover a monophyletic *Echinocactus* clade, since *E.platyacanthus* and *E.horizonthalonius* were unresolved in a trichotomy along with the *Astrophytum* clade.

***Homalocephala* clade.** This clade was recovered as monophyletic in all three concatenated analyses. Support values for this clade ranged from 90% mpb, 73% mlb and 1.0 pp in the cpDNA (Fig. 2), to 95% mpb and 0.99 pp and 99% mpb and 1.0 pp in the cpDNA plus morphology and the total evidence analyses, respectively (Fig. 3, 4). The *5GT* nuclear gene analyses also recovered the *Homalocephala* clade, although support values were lower (56% mlb and 0.72 pp) (Fig. 5). The MP analysis with *5GT* did not recover a monophyletic *Homalocephala*, since *Echinocactusparryi* was recovered as sister to the *Echinocactus* clade, but with no support. Internal relationships in the *Homalocephala* clade were unresolved in the ML and BA analyses of the cpDNA matrix recovering a polytomy of three strongly supported clades, each one integrated by three terminals of the same species (Fig. 2). The MP analysis of the cpDNA matrix recovered *E.parryi* as sister to a weakly supported clade (58% mpb) including *E.polycephalus* and *E.texensis* (Fig. 2). The sister relationship of *E.texensis* and *E.polycephalus* was weakly to highly supported in the MP (51%) and the BA (0.90 pp) analyses of the total evidence matrix and by the *5GT* nuclear analyses (73% mpb, 56 mlb and 0.99 pp) (Fig. 5). Nevertheless, the cpDNA plus morphology BA results recovered a clade including *E.parryi* and *E.polycephalus* but with low support (0.50 pp). Infraspecific resolution in the *Homalocephala* clade had low support only in the MP analysis of the cpDNA matrix, in which *E.texensis* 1 was sister to *E.texensis* 2, E.polycephalussubsp.xeranthemoides 1 was sister to E.polycephalussubsp.xeranthemoides 2 and *E.parryi* 1 was recovered as sister to *E.parryi* 2 (Fig. 2).

***Echinocactus* clade.** This clade was recovered with high support in the cpDNA plus morphology (Fig. 4) and the total evidence analyses (Fig. 3). Support values for the *Echinocactus* clade were high in the cpDNA plus morphology (87% mpb and 0.98 pp), as well as in the total evidence analyses (99% mpb and 1.0 pp). Nuclear genes analyses also recovered the *Echinocactus* clade with very high support (Fig. 5). Nevertheless, the cpDNA ML and BA results recovered a low to highly supported clade composed of *E.platyacanthus*, *E.horizonthalonius* and *Astrophytum*, although relationships were unresolved amongst all three taxa (Fig. 2). Internal resolution for *E.platyacanthus* was achieved in the cpDNA analyses, where terminal 3 was recovered as sister to a highly supported clade (97% mpb, 85% mlb and 1.0 pp) including terminals 1 and 2 (Fig. 2). Additionally, the cpDNA analyses provided infraspecific resolution for *E.horizonthalonius*, in which terminal 1 is sister to the strongly supported clade (95% mpb, 85% mlb and 0.89 pp) including terminal 2 and E.horizonthaloniussubsp.nicholii (Fig. 2).

## Discussion

**DODA and *5GT* as molecular markers in the HEA clade**. PCR amplifications for both nuclear genes were challenging, as standardisation of PCR conditions were hard to achieve due to lack of consistency even with the same protocols, reagents, concentrations, times and temperatures. It could be that the primers designed by Felker et al. (2008) have low affinity and/or that the DODA and *5GT* are low copy genes. To assess the putative orthology of these two nuclear genes, we applied the procedure of Babineau et al. (2013) ensuring that only one clear PCR band of the same length was amplified for each of the terminals and inspecting chromatograms to make sure that no double-picked nucleotides were present. Furthermore, there were no problems aligning the sequences of either of these two genes, suggesting that the analysed sequences were single copy. Other results reinforced this assumption in that endemic species with restricted distribution, such as *E.parryi*, did not show nucleotide divergence in *5GT*and DODA from individuals of different populations (Fig. 5) and that, even in different species of the clade, HEA sequence divergence was low.

Brockington et al. (2015) discovered two isoforms for DODA, the α and the β, which differ in two diagnostic amino acids at positions 183 and 227. Unfortunately, our new partial sequences were short and determination to which of the isoforms they belong was not possible. However, BLAST searches confirmed that all of our sequences are more similar to the α isoform found in a clade integrated by two species of Cactaceae, *Lophophorawilliamsii* (Lem. ex Salm-Dyck) J.M. Coult.) and *Pereskiaaculeata* Mill. and members of other families of the Caryophyllales such as Portulacaceae, Basellaceae and Talinaceae (Brockington et al. 2015). Our new sequences are more similar (97%) to the α isoform of DODA found in *Lophophorawilliamsii* (GenBank accession KR376285) than to the β isoform (70% similarity).

The phylogenetic signal of *5GT* and DODA sequences showed that these markers are useful for reconstructing evolutionary relationships in the HEA clade. Topologies obtained with the nuclear markers have good resolution and were highly congruent with those obtained with the cpDNA and cpDNA plus morphology matrices. In these regards, the topologies obtained with cpDNA plus morphology are exactly the same as those resulting from total evidence analyses (which include the nuclear dataset). The only differences between these two analyses are that, in the total evidence analyses, support for several nodes was higher. The low variability found in DODA sequences could be useful to evaluate deep phylogenetic relationships such as tribe assessments, contrary to faster evolving sequences of *5GT* that, as shown here, could also be employed for phylogeographic purposes (Fig. 5). All this allows the nuclear genome to be studied for genes related to an important and characteristic pigment pathway, such as the betalains in the Caryophyllales.

**Polyphyletic *Echinocactus*.** As well as in previous studies (Wallace 1995, Butterworth et al. 2002, Bárcenas et al. 2011), all of our analyses recovered *E.grusonii* with very high support within the *Ferocactus* clade and outside of the HEA *clade*. Nevertheless, recognition of the monotypic genus *Kroenleinia* (Lodé 2014) to include *E.grusonii* has to be evaluated more deeply, since the phylogenetic relationships in the *E.grusonii-Ferocactus* clade are still obscure (Bárcenas et al. 2011). Furthermore, several morphological characters such as epicotyl type, acute cotyledons, number of spines per areole in seedlings and seed size, are shared amongst *E.grusonii* and other members of *Ferocactus* such as *F.glaucescens*, obscuring the separation between *Kroenleinia* and *Ferocactus*.

Although the unambiguous optimisation of characters could not detect any synapomorphic characters for the *E.grusonii*-*Ferocactus* clade, deltran optimisation showed that small size seeds are a synapomorphic character for the clade. Acctran optimisation showed that a trullate shape of the floral tube scales, as in *E.grusonii*, is plesiomorphic for the *E.grusonii-Ferocactus* clade switching to ovate scales in *Ferocactus* (Fig. 3). The same pattern was found for the shape of the internal segments of the perianth where a rhombic shape, as in *E.grusonii*, was reconstructed as plesiomorphic and then they changed to a lanceolate shape in *Ferocactus* (Fig. 3). Although the tuberculate epicotyl is homoplastic, most of the species of this clade shared this state, except *F.histrix*, which has a ribbed epicotyl comparable to those in the HEA clade. The same pattern is found in the number of spines per areole at the seedling stage and with the shape of the apex of cotyledons, where species of *E.grusonii-Ferocactus* clade (except *F.histrix*) have areoles that bear 9 to 12 spines and acute cotyledons.

**HEA clade.** The close relationship between *Astrophytum* and *Echinocactus* has been well documented in previous molecular analyses (Wallace 1995, Butterworth et al. 2002, Bárcenas et al. 2011, Hernández-Hernández et al. 2011, Vázquez-Lobo et al. 2015), as well as in some morphological studies (Berger 1929, Benson 1982). This study recovered a strongly supported clade with three internal clades, *Astrophytum*, *Echinocactus* and *Homalocephala*. These results suggest that the HEA clade could be formally recognised as a clear-cut phylogenetic and taxonomic entity of North American cacti. Species of the HEA clade are characterised primarily by their pericarpel and fruit wall with narrowly triangular “pointed scales” and by the presence of trichomes, large seeds (character 34 state 3) and a disjunct configuration of the hilum-micropilar region (character 41 state 0), which the unambiguous optimisation recovered as morphological synapomorphies. As molecular synapomorphies, the analysis recovered two indels, indel 390–394 (character 51 state 0) and indel 448–456 (character 53 state 0) for the *atp*B-*rbc*L intergenic spacer (Fig. 3), which the unequivocal character optimisation identified as synapomorphies (Fig. 3). Deltran optimisation showed that character 3, number of spines per areole, state 0, (1 to 4 spines per areole at the seedling stage) is synapomorphic for the clade consisting of the *Echinocactus* and *Astrophytum* clades, but not for the *Homalocephala* clade, in which seedlings bear 5 to 8 spines (state 1) in most of taxa, except E.polycephalussubsp.xeranthemoides, which bears 9 to 12 spines per areole (Fig. 3).

So far, *K.grusonii* is the only species that has consistently been recovered outside the HEA clade having pointed scales in flowers and fruits, as well as trichomes in the axils of the scales (Wallace 1995, Butterworth et al. 2002, Bárcenas et al. 2011). Nevertheless, *K.grusonii* lacks the putative diagnostic indels for this clade found in the *atp*B-*rbc*L. Unfortunately, the parsimony reconstruction of ancestral states was ambiguous regarding the presence of pointed scales, so it is unclear whether this character is plesiomorphic or apomorphic in our phylogeny.

***Homalocephala* clade.** Phylogenetic studies conducted here strongly support the results of Wallace (1995) and the morphological study of Ferguson (1992), recognising two clades for *Echinocactus*, the *Echinocactus* and the *Homalocephala* clades. Our results disagree with those of Bárcenas et al. (2011), in which the only terminal of *Echinocactuspolycephalus* they sampled was recovered outside of the HEA clade. Here all the terminals of E.polycephalussubsp.polycephalus and E.polycephalussubsp.xeranthemoides were recovered within the *Homalocephala* clade, supporting the inclusion of these two subspecies within the genus as Wallace (1995) and Ferguson (1992) proposed.

Within the *Homalocephala* clade, the MP analysis with the cpDNA matrix, as well as the *5GT* and total evidence analyses, recovered *E.parryi* as sister to a clade with moderate support that included *E.polycephalus* and *E.texensis*. However, the ML and BA analyses of the cpDNA recovered a strongly supported polytomy for these three species (Fig. 2). Thus, more evidence is needed to fully resolve the internal relationships in the *Homalocephala* clade since our results only weekly support the sister relationship between *E.texensis* and *E.polycephalus*. Some taxonomists have elevated the rank of E.polycephalussubsp.xeranthemoides to the level of species (Rydberg 1917, Salywon and Hodgson 2012). Salywon and Hodgson (2012) proposed the recognition of *E.xeranthemoides* as a distinct species from *E.polycephalus* based on several morphological characters, such as the colour and number of spines per areole, length, colour and orientation of the fruit scales, seed length and testa ornamentation, that can easily distinguish one taxa from the other. Our hypotheses weakly supports this proposal, because these two taxa were always recovered as sisters, except in the MP analysis of the cpDNA plus morphology matrix when they were unresolved in a polytomy with the other two species of *Homalocephala* (Fig. 4). Furthermore, divergence in DNA sequences between the subspecies of *E.polycephalus* was almost null, with only three single nucleotide polymorphisms (SNP) in all the matrices analysed. However, two of these SNPs support the distinction of the two subspecies.

The only synapomorphic character detected by the unambiguous optimisation analysis for the *Homalocephala* clade was the pubescence of the abaxial face of the pericarpelar scales, which apparently was later lost in Echinocactuspolycephalussubsp.xeranthemoides (Fig. 3). However, other morphological characters support the monophyly of *Homalocephala* as compared with their most closely related genera, *Astrophytum* and *Echinocactus*. These characters are the campylotropous embryos, the cotyledons with acute apices, seedling areoles with 5 to 8 spines, acute ribs in the juvenile and the adult stages, the pubescence of the spine epidermis (except in E.polycephalussubsp.xeranthemoides), the slightly tubular flower shape and the testa cells with a channelled boundary relief.

***Echinocactus* clade.** The *Echinocactus* clade has been consistently recovered with medium to strong support in all previous phylogenetic studies (Wallace 1995, Butterworth et al. 2002, Bárcenas et al. 2011, Hernández-Hernández et al. 2011, Vázquez-Lobo et al. 2015). In our cpDNA plus morphology and total evidence analyses, this clade was recovered with high support (Fig. 3, 4). Although, the MP analysis of the cpDNA matrix also recovered a monophyletic *Echinocactus*, support values were much lower and the ML and BA analyses with this matrix instead recovered *E.platyacanthus* and *E.horizonthalonius* as unresolved in a strongly supported trichotomy including *Astrophytum* (Fig. 2). This latter result supports the ideas of Ferguson (1992) and Doweld (2000) who proposed the exclusion of *E.horizonthalonius* from the genus based on morphology alone. Furthermore, the optimisation analyses identified that an apically tuberculated epicotyl and a black-greyish testa are autapomorphic characters for *E.horizonthalonius* (Fig. 3). However, due to the recovering of a sister relationship between *E.platyacanthus* and *E.horizonthalonius* in most of the analyses developed, as well as in all previous studies, it is here preferred to maintain *E.horizonthalonius* in the genus *Echinocactus*. The unambiguous optimisation shows that the rounded apices of the cotyledons (character 2 state 2) are a synapomorphic character for the *Echinocactus* clade (Fig. 3).

The surprisingly closer phylogenetic relationship of *Echinocactus* to *Astrophytum*, rather than to *Homalocephala*, recovered in this study, is morphologically supported by two synapomorphies: the rectangular epicotyls (character 6 state 2) and a reduction from 1 to 4 spines per areole (character 3 state 0) in seedlings (Fig. 3). Acctran optimisation showed that a rectangular epicotyl is synapomorphic to this clade, which then evolved to a globose shape in *E.horizonthalonius* and *A.asterias* (Zucc.) Lem. and to a cylindrical shape in *A.caput-medusae* (Velazco & Nevárez) D.R. Hunt (Fig. 3). Deltran optimisation found that a reduction of 1 to 4 spines per areole at seedling stage and one year of age individuals is also a synapomorphic character. Thus, it seems that the *Homalocephala* clade has possibly retained plesiomorphic characters, such as campylotropous embryos, acute cotyledons and more spines per areole in seedling stages, as compared to *Astrophytum* and *Echinocactus*. These characters were modified in *Echinocactus* and *Astrophytum*, where the embryos are anatropous or orthotropous, the cotyledons rounded or obtuse and the areoles bear less spines.

Interestingly, the phylogenetic analyses here conducted recovered with medium to high support an infraspecific structure for the two widely distributed species of *Echinocactus*. It was here found that there is molecular evidence to recognise E.horizonthaloniussubsp.nicholii by the presence of various SNPs in the cpDNA alignment. There are at least two genetically well delimited populations or megapopulations of *E.platyacanthus* as Figueroa (2015) previously described (Fig. 2). This study recovered the northern populations (Tamaulipas, Coahuila and San Luis Potosí) as sister to a well-supported clade including terminals from central Hidalgo and southern Puebla (Fig. 2). Furthermore, the distinction between these two megapopulations is also supported by a deletion of 60 nucleotides in the *atp*B-*rbc*L IGS in individuals from the northern populations.

**Taxonomic implications.** The recovering of *Homalocephala* as sister to the *Echinocactus* and *Astrophytum* clade suggests that changes have to be made to the current status in order to establish a phylogenetically based classification. *Echinocactus*, as currently accepted, cannot be here recognised because this would imply the acceptance of a non-monophyletic genus since *E.grusonii* was recovered as sister to *Ferocactus* and *Homalocephala* as sister to the *Astrophytum* and *Echinocactus* clade. One possibility is to recognise the genus *Echinocactus* with three subgenera or sections (*Homalocephala, Echinocactus* and *Astrophytum*) similar to the proposal of Benson (1982). Nevertheless, the morphological diversity suggests that these clades could be easily recognised as three different genera with a balanced partition of the molecular and morphological diversities. *Astrophytum* is a morphologically well supported genus (Bravo-Hollis and Sánchez-Mejorada 1991; Anderson 2001; Vázquez-Lobo et al. 2015), with two synapomorphic characters identified in this study, pubescent epidermis (character 7, state 1) and orthotropous embryos (character 1, state 0). Although in the *Homalocephala* clade, the pubescence of the pericarpel and floral tube are the only synapomorphic characters, there are some other shared characters that support its distinction from its closest relatives of the *Astrophytum* and *Echinocactus* clade. The presence of acute cotyledons, 5 to 12 spines per areole at seedling stage, campilotropous embryos and pubescent spine epidermis in most of the species, support the recognition of *Homalocephala*. *Echinocactus* is supported by the presence of rounded cotyledons, which is a synapomorphic character for the genus. Other characters that support its distinction from its closest relatives are the campanulate flowers and the presence of a dense apical region covered with abundant trichomes. Thus, the HEA clade is here recognised as circumscribed by three clades, each one representing a genus: *Homalocephala*, *Echinocactus* and *Astrophytum*. The HEA clade could be recognised as a subtribe in the Cacteae, maybe as the Echinocactinae Buxb. (Buxbaum 1958), in which *Echinocactus* and *Homalocephala* are included. If this is the case, only the genus *Astrophytum* would have to be included to delimit a monophyletic subtribe.

## Conclusions

This is the first phylogenetic study that has evaluated and combined molecular data from chloroplast and nuclear genomes with morphology to test the monophyly of all species and subspecies of *Echinocactus* currently accepted. Here we reinforce the proposal of excluding *Echinocactusgrusonii* from the genus. Nevertheless, the recognition of *Kroenleiniagrusonii* must be deeply evaluated since phylogenetic relationships of the *Ferocactus* clade (including *K.grusonii*, *Leuchtenbergia*, *Stenocactus*, *Thelocactus* and *Glandulicactus*) are still unresolved. The well-known HEA clade was recovered as monophyletic with strong support. This clade is morphologically and molecularly well defined, suggesting its taxonomic recognition. Our results also support the proposal that *Echinocactus*, as currently accepted (excluding *K.grusonii*), should be considered as two independent lineages, the *Homalocephala* and the *Echinocactus* clades, each one with its own molecular and morphological diagnostic characters, and each one representing different genera. In this study, all of the analyses recovered *E.polycephalus* within the *Homalocephala* clade, supporting its inclusion in this taxon. Here we present the new taxonomic combinations for the species of *Homalocephala* and an identification key for the genera of the HEA clade and for all of their species and subspecies.

## Taxonomic treatment

### Homalocephala parryi

Taxon classificationPlantaeCaryophyllalesCactaceae

(Engelm.) Vargas & Bárcenas comb. nov.

urn:lsid:ipni.org:names:60477372-2

Echinocactus parryi Engelm., Proc. Amer. Acad. Arts 3: 276. 1857.

#### Type.

[Mexico], near Lake Santa Maria, Chihuahua, a bunch of spines collected by Dr. Parryi, s/n, apparently lost (**lectotype**, plate 32, figs 6–7 in Engelm., Rep. U.S. Mex. Bound., Bot. [Emory], designated by Chamberland, Syst. Bot. 22: 310. 1997).

### Homalocephala polycephala

Taxon classificationPlantaeCaryophyllalesCactaceae

(Engelm. & J.M. Bigelow) Vargas & Bárcenas comb. nov.

urn:lsid:ipni.org:names:60477373-2

Echinocactus polycephalus Engelm. & J.M. Bigelow, Proc. Amer. Acad. Arts 3: 276. 1857.

#### Type.

U.S.A., California, Mohave Valley, Bigelow s/n., 8 Mar 1854 (**lectotype**, designated by Chamberland, Syst. Bot. 22: 311. 1997: MO 2017480).

### Homalocephala polycephala subsp. xeranthemoides

Taxon classificationPlantaeCaryophyllalesCactaceae

(J.M. Coult.) Vargas & Bárcenas comb. nov.

urn:lsid:ipni.org:names:77191938-1

Echinocactus polycephalus Engelm. & J.M. Bigelow var. xeranthemoides J.M. Coult. in Contr. U.S. Natl. Herb. 3(7): 358. 1896 [1 Apr 1896].

#### Type.

U.S.A., Arizona, near the Rio Colorado, Siler s/n., Nov., 1881 (**lectotype**, designated by Benson, Cacti U. S. Canada, 951. 1982: MO 106798 [excluding packet labeled “Siler, 1882”]).

##### Key to genera of HEA clade

**Table:** 

1	Stem epidermis glabrous; ovoid to almost circular seeds; campilotropous or anatropous embryos and rounded or acute cotyledons	**2**
–	Stem epidermis pubescent; navicular seeds; orthotropous embryos and obtuse cotyledons	*** Astrophytum ***
2	Stem apex with scarce trichomes; acute ribs; slightly tubular flowers; campilotropous embryos and acute cotyledons	*** Homalocephala ***
–	Stem apex with a dense cover of trichomes; obtuse ribs; campanulate flowers, anatropous embryos and rounded cotyledons	*** Echinocactus ***

##### Key to species of *Astrophytum*

**Table:** 

1	Areoles in mature plants with conspicuous spines	**2**
–	Areoles in mature plants with inconspicuous spines	**3**
2	Stiff straight spines and completely yellow flowers	*** A. ornatum ***
–	Fexible curved spines and yellow flowers with a reddish base	*** A. capricorne ***
3	Stems ribbed	**4**
–	Stems tuberculated	*** A. caput-medusae ***
4	Stems globose to cylindrical, commonly with five acute ribs	*** A. myriostigma ***
–	Stems depressed, commonly with eight obtuse ribs	*** A. asterias ***

##### Key to species of *Homalocephala*

**Table:** 

1	Stems rarely to frequently caespitose; yellow flowers and dry fruits	**2**
–	Stems Simple; pink flowers and juicy fruits	*** H. texensis ***
2	Stems rarely caespitose; yellow flowers with a clear reddish base	*** H. parryi ***
–	Stems commonly caespitose, forming large clumps; yellow flowers without a reddish base	**3**
3	Spine epidermis and pericarpel scales pubescent; testa cells with periclinal walls with convex relief (papillated)	** H. polycephala subsp. polycephala **
–	Spine epidermis and pericarpel scales glabrous; testa cells with periclinal walls with inconspicuous relief (flat)	** H. polycephala subsp. xeranthemoides **

##### Key to species of *Echinocactus*

**Table:** 

1	Mature plants with stems large globose to barrel shaped with up to 60 ribs; yellow flowers; testa cells with inconspicuous relief	*** E. platyacanthus ***
–	Mature plants with stems depressed, globose to rarely short cylindrical frequently with eight ribs; pink to crimson flowers, seeds with testa cells with a convex relief	**2**
2	Seedlings with 1 to 2 spines per areole; mature plants with frequently depressed stems; straight spines and light pink flowers	** E. horizonthalonius subsp. horizonthalonius **
–	Seedlings commonly with 4 spines per areole; mature plants frequently with short cylindrical stems; curved spines and pink to crimson flowers	** E. horizonthalonius subsp. nicholii **

## Supplementary Material

XML Treatment for Homalocephala parryi

XML Treatment for Homalocephala polycephala

XML Treatment for Homalocephala polycephala subsp. xeranthemoides

## Appendix 1

1.1 Scientific names, voucher information and Genbank accession numbers for *atp*B-*rbc*L, *psb*A-*trn*H, *trn*L-*trn*F, *mat*K, DODA and *5GT* sequences used in the concatenated analyses. *indicates terminals with previously extracted DNA.

**Ingroup**: *Echinocactushorizonthalonius* 1, Vargas-Luna 74, QMEX, *atp*B-*rbc*L (MH129821), *psb*A-*trn*H (MH129853), *5GT* (MG149506); *trn*L-*trn*F Genbank accession: HM041258. *Echinocactushorizonthalonius* 2, Vargas-Luna 75, QMEX, *atp*B-*rbc*L (MH129820), *psb*A-*trn*H (MH129854), DODA (MG149542), *5GT* (MG149507); R. Bárcenas 1698*, QMEX, *trn*L-*trn*F (MH138285); *mat*K Genbank accession: FN997104. Echinocactushorizonthaloniussubsp.nicholii, Ecker 111, living collection of the Desert Botanical Garden, *atp*B-*rbc*L (MH129822), *psb*A-*trn*H (MH129855), *trn*L-*trn*F (MH138286), *5GT* (MG149508). *Echinocactusplatyacanthus* 1, M. Figueroa C26M6*, QMEX, *atp*B-*rbc*L (MH129817), DODA (MG149541), *5GT* (MG149505); M. Figueroa C16M1*, QMEX, *psb*A-*trn*H (MH129850), *trn*L-*trn*F (MH138282). *Echinocactusplatyacanthus* 2, M. Figueroa C9M1*, QMEX, *atp*B-*rbc*L (MH129818), *trn*L-*trn*F (MH138283); M. Figueroa C9M3*, QMEX, *psb*A-*trn*H (MH129851). *Echinocactusplatyacanthus* 3, M. Figueroa C13M1*, QMEX, *psb*A-*trn*H (MH129852); M. Figueroa C18M15*, QMEX, *atp*B-*rbc*L (MH129819), *trn*L-*trn*F (MH138284). *Homalocephalaparryi* 1, Vargas-Luna 28, QMEX, *atp*B-*rbc*L (MH129826), *psb*A-*trn*H (MH129859), DODA (MG149544), *5GT* (MG149513); *mat*K Genbank accession: KC776965. *Homalocephalaparryi* 2, Vargas-Luna 30, QMEX, *psb*A-*trn*H (MH129860); Vargas-Luna 38, QMEX, DODA (MG149545); Vargas-Luna 41, *atp*B-*rbc*L (MH129827), *5GT* (MG149514). *Homalocephalaparryi* 3, Vargas-Luna 61, QMEX, *atp*B-*rbc*L (MH129828), *5GT* (MG149515); Vargas-Luna 133, QMEX, *psb*A-*trn*H (MH129861). Homalocephalapolycephalasubsp.polycephala, M. Chamberland 51, Living collection of the Desert Botanical Garden, *atp*B-*rbc*L (MH129829), *psb*A-*trn*H (MH129862), *trn*L-*trn*F (MH138289), *5GT* (MG149519); Vargas-Luna 83, QMEX, DODA (MG149546); *mat*K Genbank accession number: FN997389. Homalocephalapolycephalasubsp.xeranthemoides 1, Vargas-Luna 180, DES, *atp*B-*rbc*L (MH129830), *psb*A-*trn*H (MH129863), *trn*L-*trn*F (MH138290), *5GT* (MG149520). Homalocephalapolycephalasubsp.xeranthemoides 2, W. Hodgson 9727, DES, *atp*B-*rbc*L (MH129831), *psb*A-*trn*H (MH129864); W. Hodgson 25463, DES, *trn*L-*trn*F (MH138291), *5GT* (MG149521). *Homalocephalatexensis* 1, Vargas-Luna 127, QMEX, *atp*B-*rbc*L (MH129823), *psb*A-*trn*H (MH129856), DODA (MG149543); Carter without number, living collection of the Desert Botanical Garden, *trn*L-*trn*F (MH138288), *5GT* (MG149512); *mat*K Genbank accession: FN997302. *Homalocephalatexensis* 2, M. Baker 14286, ASU, *atp*B-*rbc*L (MH129824), *psb*A-*trn*H (MH129857), *5GT* (MG149510). *Homalocephalatexensis* 3, M. Baker 16609, ASU, *atp*B-*rbc*L (MH129825), *psb*A-*trn*H (MH129858), *trn*L-*trn*F (MH138287), *5GT* (MG149511). *Kroenleiniagrusonii* 1, Vargas-Luna 92, QMEX, *atpB-rbcL* (MH129832), *psb*A-*trn*H (MH129865); CHB50*, living collection of Jardín Botánico Charco del Ingenio, DODA (MG149547), *5GT* (MG149523); *trn*L-*trn*F Genbank accession: HM041257; *mat*K Genbank accession: FN997149. *Kroenleiniagrusonii* 2, R. Bárcenas 1584*, QMEX, *atp*B-*rbc*L (MH129833); R. Bárcenas 1588*, QMEX, *psb*A-*trn*H (MH129866); *trn*L-*trn*F Genbank accession: HM041257.

**Outgroup**: *Astrophytumasterias*, nursey acquired plant, *atp*B-*rbc*L (MH129812), *psb*A-*trn*H (MH129845); *trn*L-*trn*F Genbank accession: KC776941; *mat*K Genbank accession: FN997357. *Astrophytumcapricorne*, H. Hernández 2320*, MEXU, *atp*B-*rbc*L (MH129816), *psb*A-*trn*H (MH129846), *trn*L-*trn*F Genbank accession: KC776959, *mat*K Genbank accession: FN997272*Astrophytumcaput-medusae*, nursery acquired plant*, *atp*B-*rbc*L (MH129813), *psb*A-*trn*H (MH129847); *trn*L-*trn*F Genbank accession: KC776998; *matK* Genbank accession: FN997077. *Astrophytummyriostigma*, Vargas-Luna 116, QMEX, *atp*B-*rbc*L (MH129814), *psb*A-*trn*H (MH129848), DODA (MG149538); *trn*L-*trn*F Genbank accession: KC776951; *mat*K Genbank accession: FN997366. *Astrophytumornatum*, Vargas-Luna 95, QMEX, *atp*B-*rbc*L (MH129815), *psb*A-*trn*H (MH129849), DODA (MG149540); H. Hernández 3703*, MEXU, *5GT* (MG149504); *trn*L-*trn*F Genbank accession: KC776947; *mat*K Genbank accession: FN997293. *Aztekiumhintonii*, ZSS991236*, Living collection Zurich Botanical Garden, *atp*B-*rbc*L (MH129810), *psb*A-*trn*H (MH129843), DODA (MG149537); *mat*K Genbank accession: FN997040. *Aztekiumritteri*, nursery acquired plant, *atp*B-*rbc*L (MH129811), *psb*A-*trn*H (MH129844); *mat*K Genbank accession: AY015290. *Carnegieagigantea*, Vargas-Luna 86, QMEX, *atp*B-*rbc*L (MH129808), DODA (MG149536), *5GT* (MG149503); *trn*L-*trn*F Genbank accession: HM041236; *mat*K Genbank accession: KT164775. *Ferocactusechidne*, R. Bárcenas 49*, MEXU, *atp*B-*rbc*L (MH129835), *psb*A-*trn*H (MH129868), *5GT* (MG149530); *trn*L-*trn*F Genbank accession: HM041276; *mat*K Genbank accession: FN997544. *Ferocactusglaucescens*, H. Hernández 3701*, MEXU, *atp*B-*rbc*L (MH129834), *5GT* (MG149524); H. Hernández 2425*, MEXU, *psb*A-*trn*H (MH129867); *mat*K (FN997274). *Ferocactushamatacanthus*, H. Hernández 2096*, MEXU, *atp*B-*rbc*L (MH129836), *5GT* (MG149531); H. Hernández 1467*, MEXU, *psb*A-*trn*H (MH129870); *mat*K Genbank accession: FN997248. *Ferocactushistrix*, J. Álvarez SLP1*, QMEX, *atp*B-*rbc*L (MH129837), DODA (MG149549), *5GT* (MG149526); R. Bárcenas 557*, MEXU, *psb*A-*trn*H (MH129869); *mat*K Genbank accession: FN997287. *Ferocactuslatispinus*, Vargas-Luna 90, QMEX, *atpB-rbcL* (MH129838); J. Álvarez without number*, QMEX, *psb*A-*trn*H (MH129871); R. Bárcenas 1620*, QMEX, *5GT* (MG149532); *trn*L-*trn*F Genbank accession: HM041278; *mat*K Genbank accession: FN997246. *Ferocactuslindsayi*, CHB10*, living collection of Jardín Botánico Charco del Ingenio, *atp*B-*rbc*L (MH129839), *5GT* (MG149533); *mat*K Genbank accession: FN997152. *Ferocactuspilosus*, C. Gómez-Hinostrosa 739*, MEXU, *atp*B-*rbc*L (MH129840), *psb*A-*trn*H (MH129872), *5GT* (MG149534); *mat*K Genbank accession: FN997133. *Ferocactuswislizeni*, R. Bárcenas 1565*, QMEX, *psb*A-*trn*H (MH129873), *5GT* (MG149535); R. Bárcenas 1569*, QMEX, *atp*B-*rbc*L (MH129841); *mat*K Genbank accession: FN997459. *Geohintoniamexicana*, Reyes J. 3226*, MEXU, *atp*B-*rbc*L (MH129809), *psb*A-*trn*H (MH129842), *mat*K GenBank accession: FN997326. *Opuntiaficus-indica*, Genbank accessions: *atp*B-*rbc*L JF787222, *psb*A-*trn*H FJ026612, *trn*L-*trn*F KJ735936, *mat*K FN997314, DODA EU0899741, *5GT*EU089744.

1.2 Scientific names, voucher information and Genbank accession numbers for the *5GT* nuclear gene analyses. * indicates terminals with previously extracted DNA.

**Ingroup**: *Echinocactushorizonthalonius* 1, Vargas-Luna 74, QMEX, (MG149506). *Echinocactushorizonthalonius* 2, Vargas-Luna 75, QMEX, (MG149507). Echinocactushorizonthaloniussubsp.nicholii, Ecker 111, living collection of the Desert Botanical Garden, (MG149508). *Echinocactushorizonthalonius* x *platyacanthus*, W. Hodgson without number, ASU, (MG149509). *Echinocactusplatyacanthus*, M. Figueroa C26M6*, QMEX, (MG149505). *Homalocephalaparryi* 1, Vargas-Luna 28, QMEX, (MG149513). *Homalocephalaparryi* 3, Vargas-Luna 41, QMEX, (MG149514). *Homalocephalaparryi* 4, Vargas-Luna 61, QMEX, (MG149515). *Homalocephalaparryi* 5, Vargas-Luna 64, QMEX, (MG149516). *Homalocephalaparryi* 6, Vargas-Luna 72, QMEX, (MG149517). Homalocephalapolycephalasubsp.polycephala 1, E. Anderson 6191, living collection of the Desert Botanical Garden, (MG149518). Homalocephalapolycephalasubsp.polycephala 2, M. Chamberland 51, Living collection of the Desert Botanical Garden, (MG149519). Homalocephalapolycephalasubsp.xeranthemoides 1, W. Hodgson 30366, DES, (MG149522). Homalocephalapolycephalasubsp.xeranthemoides 2, W. Hodgson 25463, DES, (MG149521). Homalocephalapolycephalasubsp.xeranthemoides 3, Vargas-Luna 180, DES, (MG149520). *Homalocephalatexensis* 1, M. Baker 14286, ASU, (MG149510). *Echinocactustexensis* 2, M. Baker 16609, ASU, (MG149511). *Homalocephalatexensis* 3, Carter without number, living collection of the Desert Botanical Garden, (MG149512). *Kroenleiniagrusonii*, CHB50*, living collection of Jardín Botánico Charco del Ingenio, (MG149523).

**Outgroup**: *Amaranthustricolor*, Genbank accession: KP174810. *Astrophytumornatum* 1, H. Hernández 3703*, MEXU, (MG149504). *Beta vulgaris*, Genbank accession: AY526080. *Carnegieagigantea*, Vargas-Luna 86, QMEX, (MG149503). *Ferocactusechidne*, R. Bárcenas 49*, MEXU, (MG149530). *Ferocactusglaucescens*, H. Hernández 3701*, MEXU, (MG149524). *Ferocactushamatacanthus*, H. Hernández 2096*, MEXU, (MG149531). *Ferocactushistrix* 1, J. Álvarez B1*, QMEX, (MG149529). *Ferocactushistrix* 2, J. Álvarez B2*, QMEX. *Ferocactushistrix* 3, J. Álvarez SLP1*, QMEX, (MG149526). *Ferocactushistrix* 4, J. Álvarez SLP3*, QMEX, (MG149527). *Ferocactushistrix* 5, J. Álvarez SLP6*, QMEX, (MG149528). *Ferocactushistrix* 6, J. Álvarez T8*, QMEX. *Ferocactushistrix* 7, J. Álvarez T10*, QMEX, (MG149525). *Ferocactuslatispinus*, R. Bárcenas 1620*, QMEX, (MG149532). *Ferocactuslindsayi*, CHB10*, living collection of Jardín Botánico Charco del Ingenio, (MG149533). *Ferocactuspilosus*, C. Gómez-Hinostrosa 739*, MEXU, (MG149534). *Ferocactuswislizeni*, R. Bárcenas 1565*, QMEX, (MG149535). *Opuntiaficus-indica* 3, Genbank accession: EU089744, *Opuntiaficus-indica* 4, Genbank accession: EU089743.

## Appendix 2

**Table A1.** Morphological characters, character states, and development stages at which the characters were coded. **A**. Embryo, **B**. One week to one month seedlings, **C**. One year individuals, **D**. Juvenile, **E**. Adult, **F**. Adult flower, **G**. Adult fruit, and **H**. Seed. Numbers in parenthesis of indels are the positions at which they are located in the total alignment.

**Characters and states**	**Development stages**
**1) Embryo type**, 0 = orthotropous; 1 = anatropous; 2 = campylotropous	A
**2) Cotyledon apex**, 0 = acute; 1 = obtuse; 2 = rounded	B
**3) Number of spines per areole**, 0 = 1 to 4; 1 = 5 to 8; 2 = 9 to 12	B
**4) Presence of cotyledons**, 0 = inconspicuous; 1 = conspicuous	C
**5) Epicotyl type**, 0 = totally tuberculate; 1 = ribbed; 2 = apically tuberculate	C
**6) Epicotyl shape**, 0 = globose; 1 = cylindrical; 2 = rectangular	C
**7) Epidermis**, 0 = glabrous; 1 = pubescent	C
**8) Growth form**, 0 = simple; 1 = cespitose	E
**9) Stem colour**, 0 = light green; 1; dark green	E
**10) Epidermis**, 0 = glabrous; 1 = pubescent	E
**11) Stem apex**, 0 = glabrous; 1 = slightly cover with wool; 2 = densely cover with wool	E
**12) Rib shape**, 0 = acute; 1 = obtuse	D, E
**13) Distal portion of the ribs**, 0 = undulated; 1 = straight	E
**14) Spine epidermis**, 0 = glabrous; 1 = pubescent	E
**15) Trichomes length in reproductive areoles**, 0 = small; 1 = large	E
**16) Flower shape**, 0 = campanulate; 1 = slightly tubular	F
**17) Pericarpel wall**, 0 = naked; 1 = with accessory appendages	F
**18) Type of accessory appendages of the pericarpel**, 0 = scale; 1 = spine	F
**19) Presence of trichomes in flower areoles**, 0 = absent; 1 = present	F
**20) colour of the pericarpel in anthesis**, 0 = green; 1 = light green; 2 = whitish with pink; 3 = pink to purple; 4 = brownish	F
**21) Pericarpel scale shape**, 0 = semicircular; 1 = narrowly triangular “pointed scales”	F
**22) Pericarpel scale margin**, 0 = entire; 1 = Serrate	F
**23) Pericarpel scale apex**, 0 = acuminate; 1 = mucronate	F
**24) Abaxial surface of the pericarpel scales**, 0 = glabrous; 1 = pubescent	F
**25) Floral tube scale shape**, 0 = triangular; 1 = ensiform; 2 = trullate; 3 = ovate	F
**26) Perianth external segments shape**, 0 = obovate; 1 = oblanceolate; 2 = elliptic; 3 = oblong; 4 = lanceolate	F
**27) Perianth internal segments shape**, 0 = cordate; 1 = obovate; 2 = oblanceolate; 3 = elliptic; 4 = rhombic; 5 = lanceolate	F
**28) Fruit type**, 0 = dried; 1 = fleshy juicy	G
**29) Fruit wall**, 0 = naked; 1 = with accessory appendages	G
**30) Spine presence in fruit**, 0 = absent; 1 = present	G
**31) Trichomes presence in fruit**, 0 = absent; 1 = present	G
**32) Scale presence in fruit**, 0 = absent; 1 = presence	G
**33) Scale shape of fruit**, 0 = ovate; 1 = narrowly triangular “pointed scales”	G
**34) Seed size**, 0 = very small; 1 = small; 2 = medium; 3 = large; 4 = very large; 5 = extremely large	H
**35) Position of the hilum with respect to the long axis**, 1 = perpendicular; 2 = oblique; 3 = parallel	H
**36) Colour of the testa**, 0 = black; 1 = black-brown; 2 = reddish; 3 = black-grey	H
**37) Keel presence in seed**, 0 = absent; 1 = presence	H
**38) Boundary relief in testa cells**, 0 = inconspicuos; 1 = channeled; 2 = raised	H
**39) Relief of the periclinal walls of the testa cells**, 0 = none (flat); 1 = convex; 2 = concave; 3 = partially convex; 4 = partially concave	H
**40) Microrelief of the testa cells**, 0 = glabrous; 1 = verrucose; 2 = striate	H
**41) Hilum-micropyle configuration**, 0 = disjunct; 1= conjunct but separated by sclerified tissue; 2 = fused	H
**42) Presence of appendages**, 0 = absent; 1 = present	H
**43) Indel**, (54-55)	–
**44) Indel**, (58-59)	–
**45) Indel**, (82-85)	–
**46) Indel**, (122)	–
**47) Indel**, (161-169)	–
**48) Indel**, (186-191)	–
**49) Indel**, (381)	–
**50) Indel**, (388-389)	–
**51) Indel**, (390-394)	–
**52) Indel**, (419-423)	–
**53) Indel**, (448-456)	–
**54) Indel**, (517-541)	–
**55) Indel**, (572-576)	–
